# Association between acute myocardial infarction-to-cardiac rupture time and in-hospital mortality risk: a retrospective analysis of multicenter registry data from the Cardiovascular Research Consortium-8 Universities (CIRC-8U)

**DOI:** 10.1007/s00380-020-01762-2

**Published:** 2021-01-16

**Authors:** Kihei Yoneyama, Yuki Ishibashi, Yorihiko Koeda, Tomonori Itoh, Yoshihiro Morino, Takao Shimohama, Junya Ako, Yuji Ilari, Koichiro Yoshioka, Tomoyuki Kunishima, Shu Inami, Tetsuya Ishikawa, Hiroyuki Sugimura, Ken Kozuma, Keiki Sugi, Hideaki Yoshino, Yoshihiro J. Akashi

**Affiliations:** 1 Division of Cardiology, Department of Internal Medicine, St. Marianna University School of Medicine, 2-16-1, Sugao, Miyamae-ku, Kawasaki-City, Kanagawa 216-8511 Japan; 2 Division of Cardiology, Department of Internal Medicine, Iwate Medical University, Morioka, Japan; 3 Department of Cardiovascular Medicine, Kitasato University School of Medicine, Sagamihara, Japan; 4 Division of Cardiology, Tokai University School of Medicine, Isehara, Japan; 5 Department of Cardiovascular Medicine, Dokkyo Medical University, Mibu, Japan; 6 Department of Cardiology, Saitama Medical Center, Dokkyo Medical University, Koshigaya, Japan; 7 Division of Cardiology, Nikko Medical Center, Dokkyo Medical University, Nikko, Japan; 8 Division of Cardiology, Department of Internal Medicine, Teikyo University, Tokyo, Japan; 9 Division of Cardiology, Saitama Medical University International Medical Center, Saitama, Japan; 10 Department of Cardiology, Kyorin University School of Medicine, Tokyo, Japan

**Keywords:** Myocardial infarction, Cardiac rupture, Mortality, Mechanical complications, Surgery

## Abstract

Despite the known association of cardiac rupture with acute myocardial infarction (AMI), it is still unclear whether the clinical characteristics are associated with the risk of in-hospital mortality in patients with AMI complicated by cardiac rupture. The purpose of this study was to investigate the association between the time of cardiac rupture occurrence and the risk of in-hospital mortality after AMI. We conducted a retrospective analysis of multicenter registry data from eight medical universities in Eastern Japan. From 10,278 consecutive patients with AMI, we included 183 patients who had cardiac rupture after AMI, and examined the incidence of in-hospital deaths during a median follow-up of 26 days. Patients were stratified into three groups according to the AMI-to-cardiac rupture time, namely the > 24-h group (*n* = 111), 24–48-h group (*n* = 20), and < 48-h group (*n* = 52). Cox proportional hazards regression analysis was used to estimate the hazard ratio (HR) and the confidence interval (CI) for in-hospital mortality. Around 87 (48%) patients experienced in-hospital death and 126 (67%) underwent a cardiac surgery. Multivariable Cox regression analysis revealed a non-linear association across the three groups for mortality (HR [CI]; < 24 h: 1.0, reference; 24–48 h: 0.73 [0.27–1.86]; > 48 h: 2.25 [1.22–4.15]) after adjustments for age, sex, Killip classification, percutaneous coronary intervention, blood pressure, creatinine, peak creatine kinase myocardial band fraction, left ventricular ejection fraction, and type of rupture. Cardiac surgery was independently associated with a reduction in the HR of mortality (HR [CI]: 0.27 [0.12–0.61]) and attenuated the association between the three AMI-to-cardiac rupture time categories and mortality (statistically non-significant) in the Cox model. These data suggest that the AMI-to-cardiac rupture time contributes significantly to the risk of in-hospital mortality; however, rapid diagnosis and prompt surgical interventions are crucial for improving outcomes in patients with cardiac rupture after AMI.

## Introduction

Cardiac rupture associated with acute myocardial infarction (AMI) is a rare but critical condition [1–5]. Left ventricular free wall rupture can rapidly degenerate into a cardiac tamponade and can lead to death. Various epidemiological studies on predictors for cardiac rupture have reported that it is more likely to occur in the elderly, women, individuals with hypertension, and in patients who experience their first AMI [2–5].

Due to it being a rare disease, there is little knowledge of the factors associated with prognosis among patients who have had a cardiac rupture. For example, some cardiac ruptures occur during the acute phase of myocardial infarction, while others occur a few days or weeks later. However, the relationship between the time of onset of cardiac rupture and its prognosis is unclear. We believe that surgical repair is effective, but because of the rarity of the disease, randomized studies are difficult to perform. Therefore, there is a lack of evidence that repair procedures reduce the risk of death in patients with cardiac rupture.

To investigate the relationship between cardiac rupture and death, we conducted a retrospective analysis of multicenter registry data in Japan. This registry study aimed to investigate a rare disease and included eight universities across eastern Japan. Although the widespread use of percutaneous coronary interventions has reduced the frequency of cardiac ruptures in the recent years [1, 6, 7], clinicians need to have knowledge and evidence of the risk factors of such a rare condition. The purpose of this study was to investigate the association between the time of occurrence of cardiac rupture and the risk of in-hospital mortality after AMI. The results of this study may provide important insights into the prognostic value of clinical indicators associated with AMI.

## Materials and methods

### Data collection

The Cardiovascular Research Consortium-8 Universities (CIRC-8U) is a retrospective study designed to investigate the associations that underlie the complications and treatment of cardiovascular diseases among symptomatic patients [8, 9]. The CIRC-8U included eight university hospitals or their branch hospitals in eastern Japan, namely the Iwate Medical University, Kitasato University Hospital, Tokai University Hospital, Dokkyo Medical University Saitama Medical Center (Koshigaya Hospital), Dokkyo Medical University Nikko Medical Center, Saitama Medical University International Medical Center, St. Marianna University Hospital, Teikyo University Hospital, and Kyorin University Hospital. This study was conducted in accordance with the code of ethics stated in the Declaration of Helsinki after receiving approval from the ethics committee of each institute. Clinical information, including patient characteristics, was obtained from the medical records. Patient consent was not needed due to the retrospective nature of the study.

The CIRC-8U collected data on 235 patients with AMI who experienced cardiac ruptures between May 1, 1997 and December 31, 2014 (i.e., 2.3% of the 10,278 AMI patients actually encountered). The database included data on the following elements: demographics for each patient (age, gender, smoking, and medical history), demographics for AMI on admission (Killip classification; body mass index; blood pressure and heart rate; levels of serum creatinine, hemoglobin A1c, total cholesterol, high-density lipoprotein cholesterol (HDL-C), and low-density lipoprotein cholesterol (LDL-C); max creatine kinase myocardial band (CK-MB); left ventricular ejection fraction; and emergency coronary angiography data), data on cardiac rupture (septal rupture, free wall rupture, papillary muscle rupture, and time for rupture), and data on treatment (PCI and repaired surgery). The AMI-to-cardiac rupture time was calculated by subtracting the time of cardiac rupture from the time of AMI onset. Onset time was determined by a cardiologist as the time of acute occurrence of chest pain.

Of the 235 patients assessed for eligibility, 52 were excluded due to missing data on the AMI-to-rupture time. Thus, a total of 183 patients were finally included in the analysis.

### Outcomes

The primary outcome was in-hospital mortality. Patients were stratified into three groups according to the AMI-to-cardiac rupture time: the > 24-h group (*n* = 111), the 24–48-h group (*n* = 20), and the < 48-h group (*n* = 52).

### Statistical analysis

All continuous variables are expressed as medians and interquartile ranges (i.e., the differences between the 25th and 75th percentiles). Nonparametric Wilcoxon-type trend tests were used to test the trends of continuous variables across the three groups, while *χ*^2^ tests were used to evaluate the intergroup differences in the categorical variables.

We used Cox proportional hazards regression analysis to estimate the hazard ratios (HRs) and 95% confidence intervals (CIs) for in-hospital deaths according to the three groups after adjusting for covariates. The Cox models were adjusted for age, sex, Killip classification, emergent percutaneous coronary intervention, systolic blood pressure, log-transformed serum creatinine, log-transformed CK-MB, left ventricular ejection fraction, type of cardiac rupture, and repair surgery. All analyses were performed using STATA statistical software version 14 (Stata Corp, College Station, TX, USA).

## Results

A total of 183 patients with cardiac rupture after AMI (median age: 75 years, percentage of men: 54%) were examined for the association of the AMI-onset-to-rupture time with the in-hospital death events (*n* = 87, 48%) and cardiac repair surgery (*n* = 126, 67%) during a median duration of 26 days.

**Fig. 1:**
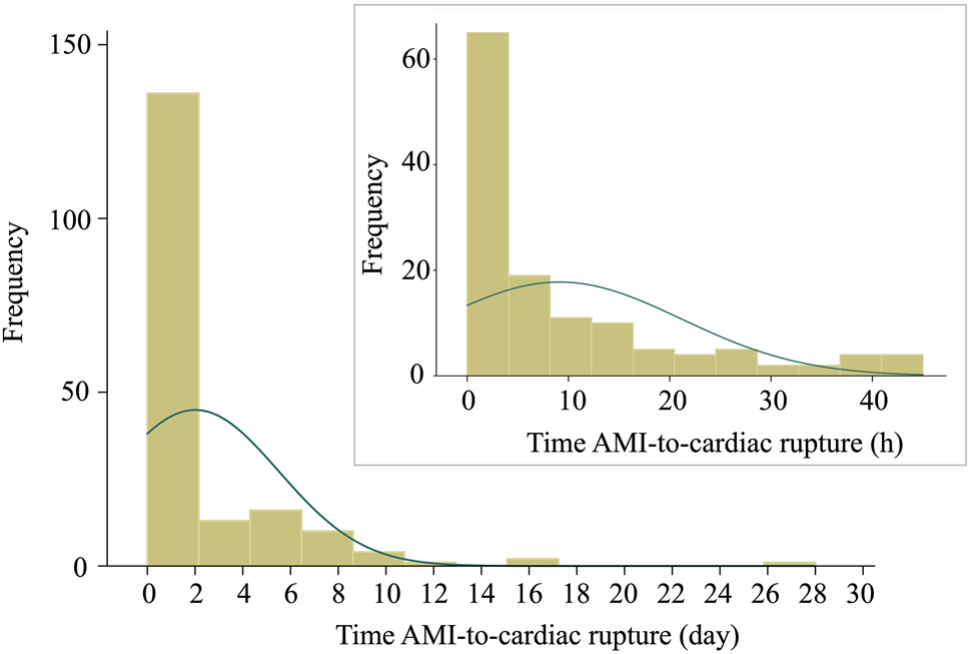
Frequency distribution of in-hospital deaths among patients with cardiac rupture after acute myocardial infarction. Cardiac rupture is common during the early stages following the onset of myocardial infarction. The distribution showed a positive skew. Data collected within 48 h are highlighted separately in the box on the right

The baseline characteristics according to the AMI-to-rupture time groups are presented in Table 1. Hypertension was more common in the > 48-h group. The body mass index, systolic blood pressure, diastolic blood pressure, and LDL level tended to be progressively higher across groups with increasing AMI-to-cardiac rupture time (all, *p* for trend < 0.05). The Killip class was progressively lower across groups with increasing AMI-to-cardiac rupture time (*p* for trend = 0.005). No trends were noted in the peak CK-MB fraction and the left ventricular ejection fraction with increasing AMI-to-cardiac rupture time. The prevalence of ST elevation MI, septal rupture, free wall rupture, and papillary muscle rupture did not differ among the three groups. Repair surgery was common in the 24–48-h group (*p* = 0.001). The frequency distribution of the in-hospital deaths among patients with cardiac rupture after AMI is shown in Fig. 1. Cardiac rupture was more frequent within 48 h of MI onset, and even within these groups (> 24-h and 24–48-h groups), cardiac rupture was more common during the early stages of AMI.

**Table 1:** Baseline characteristics of the patients according to the AMI-to-rupture time

	AMI-to-cardiac rupture time: < 24 h (*n* = 111)	AMI-to-cardiac rupture time: 24–48 h (*n* = 20)	AMI-to-cardiac rupture time: > 48 h (*n* = 52)	Data missing	*p* for trend
Age, years	75 (11)	75 (9)	75 (15)	1	0.528
Men, *n* (%)	54 (49)	9 (45)	28 (54)	0	0.747
Current smoker, *n* (%)	44 (44)	7 (35)	30 (60)	13	0.087
Hypertension, *n* (%)	63 (57)	15 (75)	42 (81)	0	0.007
Diabetes mellitus, *n* (%)	32 (29)	12 (60)	9 (17)	0	0.002
Body mass index, kg/m^2^	22 (4)	24 (5)	24 (4)	12	0.006
Emergency department					
Killip classification	3 (3)	3 (3)	1 (2)	3	0.005
STEMI, *n* (%)	105 (95)	17 (85)	49 (94)	0	0.270
Systolic BP, mmHg	108 (38)	102 (52)	124 (35)	6	0.002
Diastolic BP, mmHg	68 (31)	61 (30)	80 (22)	5	0.001
Heart rate, bpm	100 (31)	92 (20)	93 (35)	1	0.922
Creatinine, mg/dL	1 (1)	1 (1)	1 (1)	2	0.168
Hemoglobin A1c, %	6 (1)	7 (1)	6 (1)	41	0.162
Total cholesterol, mg/dL	170 (51)	199 (67)	181 (55)	23	0.310
High-density lipoprotein cholesterol, mg/dL	46 (18)	46 (24)	40 (28)	54	0.408
Low-density lipoprotein cholesterol, mg/dL	105 (49)	113 (73)	123 (47)	49	0.016
Left ventricular ejection fraction, %	47 (21)	50 (12)	45 (13)	21	0.409
Emergency CAG, *n* (%)	94 (85)	19 (95)	46 (88)	0	0.419
Primary PCI, *n* (%)	35 (32)	10 (50)	27 (52)	0	0.027
max CK-MB, U/L	114 (232)	201 (385)	158 (284)	12	0.684
Types of mechanical complications after AMI					
Repair surgery, *n* (%)	80 (72)	19 (95)	27 (52)	0	0.001
Septal rupture, *n* (%)	47 (42)	10 (50)	17 (33)	0	0.329
Free wall rupture, *n* (%)	68 (61)	13 (65)	32 (62)	0	0.950
Papillary muscle rupture, *n* (%)	3 (3)	0 (0)	5 (10)	0	0.079

Values are expressed as medians (interquartile range)

*AMI* acute myocardial infarction, *BP* blood pressure, *CAG* coronary angiography, *CK-MB* creatinine kinase myocardial band, *PCI* percutaneous coronary intervention, *STEMI* ST segment elevation myocardial infarction

### Incidence of in-hospital death after cardiac rupture

**Fig. 2:**
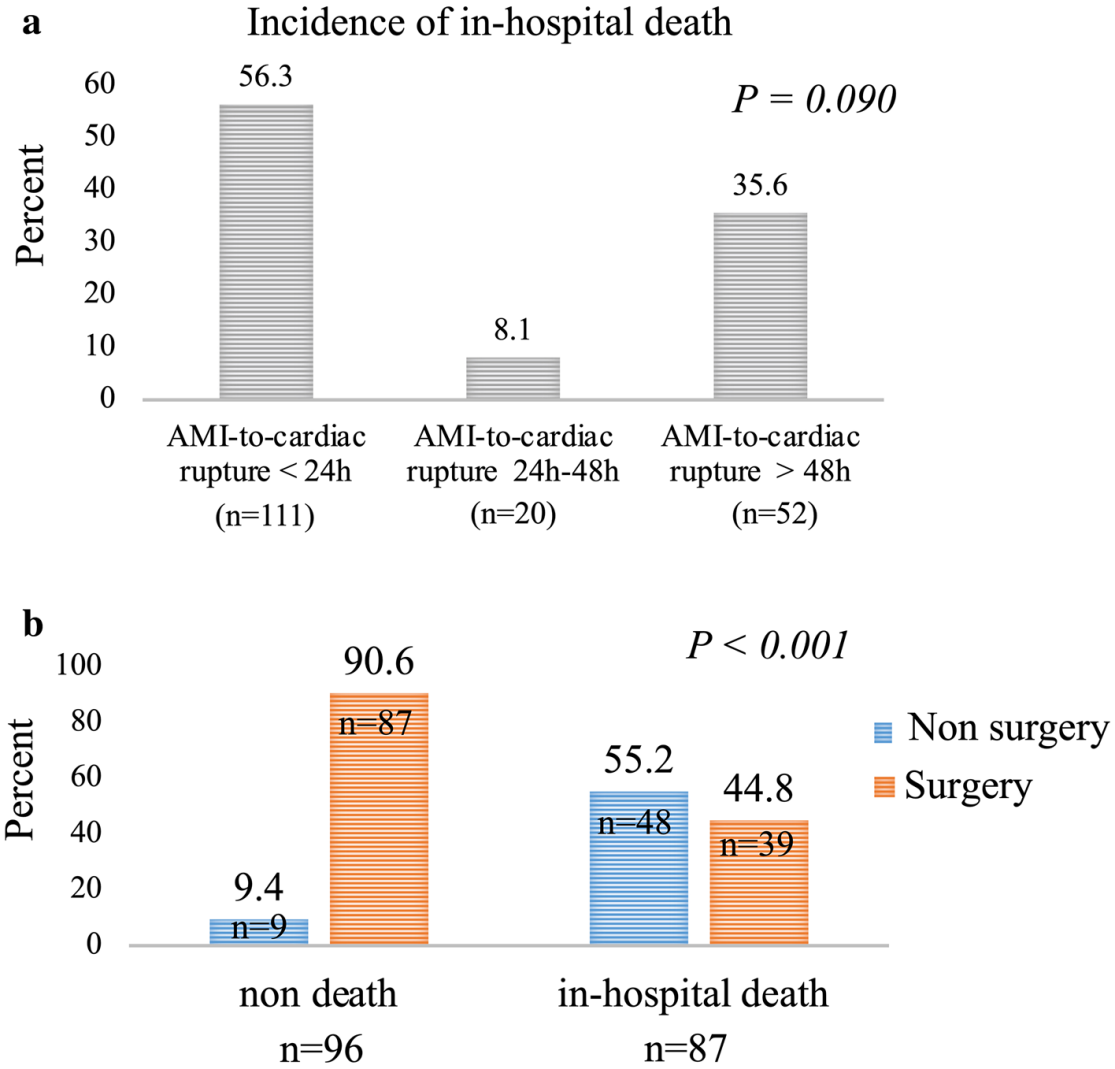
Incidence of in-hospital death after cardiac rupture. **a** The relationship between in-hospital mortality and the AMI group was non-linear. **b** Patients who underwent cardiac repair were more likely to have survived, and those who did not undergo cardiac repair were more likely to have experienced in-hospital death

The prevalence of in-hospital deaths in the > 24-h, 24–48-h, and 48-h groups were 56.3%, 8.1% and 35.6%, respectively (Fig. 2a). Of the 96 survivors after cardiac rupture, 90.6% underwent a repair surgery, while 9.4% did not (Fig. 2b). Of the 87 in-hospital deaths after cardiac rupture, 55.2% did not undergo repair surgery prior to death.

**Fig. 3:**
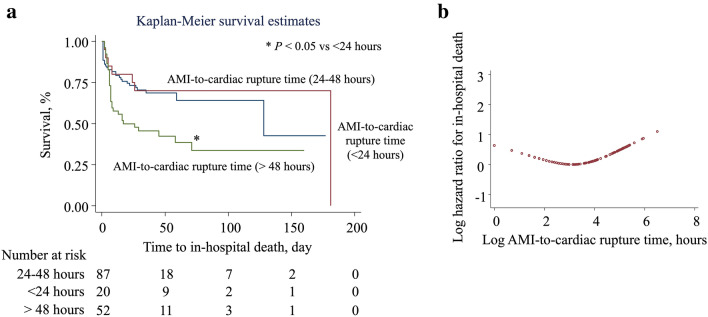
AMI-to-cardiac rupture time and the risk of in-hospital mortality among patients with cardiac rupture after AMI. **a** Kaplan–Meier estimates of survival indicated that the > 48-h group had a significantly lower survival rate than the 24–48-h group (*p* < 0.05). **b** Cox proportional hazard regression analysis was used to estimate the unadjusted hazard ratio of the relationship between the AMI-to-cardiac rupture time and the incidence of in-hospital death using a restricted cubic spline with 3 knots (1.6 [4.9], 3.3 [27], and 5.1 [164] h) using STATA. *AMI* acute myocardial infarction

The Kaplan–Meier estimates of survival are displayed in Fig. 3a. The > 48-h group had a significantly lower survival rate than the 24–48-h group (*p* < 0.05). The association between the risk of in-hospital death and the AMI-to-cardiac rupture time was non-linear (Fig. 3b).

Multivariable Cox regression analysis (Table 2 and Fig. 3) indicated a non-linear association across the three groups for mortality (HR [CI]; < 24-h: 1.0, reference; 24–48-h: 0.73 [0.27–1.86]; > 48-h: 2.25 [1.22–4.15]) after adjustments for age, sex, Killip classification, percutaneous coronary intervention, blood pressure, creatinine, peak CK-MB fraction, left ventricular ejection fraction, and type of rupture (Model 1). With the addition of cardiac repair surgery in Model 1, repair surgery was independently associated with a reduction in the HR of mortality (HR [CI]: 0.27 [0.12–0.61]) and attenuated the association between the three AMI-to-cardiac rupture time categories and the in-hospital death (statistically non-significant) in the Cox model (Model 2).

**Table 2:** The association of AMI-to-cardiac rupture time with in-hospital death

	Cox proportional hazard ratio (95% CI)	
	Model 1^a^ (risk)	Model 2^b^ (risk and surgery)
AMI-to-cardiac rupture time: < 24 h	Reference	Reference
AMI-to-cardiac rupture time: 24–48 h	0.70 (0.27–1.86)	0.82 (0.30–2.22)
AMI-to-cardiac rupture time: > 48 h	2.25 (1.22–4.15)*	1.36 (0.66–2.80)
Surgery	–	0.27 (0.12–0.61)*

^a^Model 1 included age, gender, Killip classification, primary percutaneous coronary intervention, systolic blood pressure, log-transformed serum creatinine, log-transformed CK-MB, left ventricular ejection fraction, the type of cardiac rupture (the septal wall or the free wall of the left ventricle), and presence of a papillary muscle rupture

^b^Model 2 included surgery in addition to the parameters included in Model 1

^*^p < 0.05

## Discussion

Cardiac rupture has a very high mortality; however, the association between the time of its occurrence and the subsequent prognosis is unknown. To investigate the relationship between cardiac rupture and death, we included 183 patients in our study who experienced cardiac rupture after AMI, and examined the incidence of in-hospital deaths among them. We found that: (1) cardiac rupture occurred more frequently within 48 h of MI onset; patients with an AMI-to-cardiac rupture time < 24 h in particular had a higher risk of in-hospital mortality, (2) while cardiac rupture was less frequent after more than 48 h had lapsed after AMI, it had a higher risk of in-hospital mortality, and (3) cardiac repair surgery was associated with lower in-hospital mortality, independent of the AMI-to-cardiac rupture time. The results of this study may provide important insights into the prognostic value of clinical indicators associated with AMI. Making a quick diagnosis and initiating prompt surgical interventions are crucial to improve the outcomes in patients with cardiac rupture after AMI.

We found that cardiac rupture was more common within 48 h after AMI and was less common after more than 48 h had lapsed. Van Tassel et al. [10] studied 40 cases of ruptured MI, and found that most ruptures (63%) occurred within 4 days of AMI onset; two cases experienced ruptures within 14 days of AMI onset, which was similar to our distribution. In the most recent Chinese single-center report, 75 cardiac ruptures occurred within 4 h to 17 days from AMI onset, with a mean onset time of 4.4 days [11]. Although the time of onset tended to be slightly delayed as compared to our observations, the tendency for early cardiac rupture occurrence was similar in both studies. Cardiac rupture after AMI was found to be more common during the very acute phase of the onset, and rarely occurred more than a week after the onset.

However, compared to previous observational studies, we found that AMI-to-cardiac rupture times of < 24 h and > 48 h had a higher risk of in-hospital mortality. The potential mechanisms underlying the high risk of death among patients with AMI-to-cardiac rupture times of < 24 h and > 48 h have not been clarified. Fang et al. [12] have elucidated the pathogenesis of myocardial rupture by examining mice who experienced myocardial rupture after AMI. They observed that inflammatory cell infiltration following acute MI markedly increased the regional matrix metalloproteinase (MMP) activity, leading to a rapid degradation of collagen fibrils. This damage to the fibrillar collagen in the extracellular matrix may cause significant thinning of the infarcted wall and regional dilatation, indicating infarct expansion in the super acute phase [13]. Gao et al. [14] conducted experiments to stretch the myocardium in mice with MI, and examined the relationship between the myocardial strength force and the time of cardiac rupture onset; they found that the strength force in the myocardium was low up to 3 days after the onset of MI. However, this strength force improved to normal levels 1 week after the onset of AMI. These results suggest that in the hyperacute phase, damage to the extracellular matrix and collagen fibers due to excessive inflammation can reduce the myocardial tensile strength and lead to cardiac rupture. Fang et al. [12] also demonstrated that cardiac rupture after 1 week indicated that there may be a delay in the myocardial healing process. Therefore, we thought that the relationship between AMI onset time and cardiac rupture may be seen as a difference in the pathology between the acute and chronic phases of AMI.

We found that cardiac repair surgery was associated with a lower in-hospital mortality, independent of the AMI-to-cardiac rupture time. Mantovani et al. [15] reported that 17 patients underwent repair surgery for cardiac free wall rupture after MI, and three (17.6%) died in the hospital during the surgery. Analysis of the data from the Society of Thoracic Surgeons database (USA) to identify adults (aged > 18 years) who underwent post-MI ventricular septal defect repair between 1999 and 2010 revealed that the overall operative mortality was 42.9%, and the highest operative mortality was among patients who underwent a ventricular septal defect repair within 6 h from MI. The mortality for repair surgery is relatively high, but it was suggested that the surgery may have reduced mortality among patients with cardiac ruptures associated with a very high mortality, an observation which supports our findings.

Our study did not include patients with MI without cardiac rupture and could not determine which factors would prevent rupture. However, our data indicated that prompt diagnosis and prompt initiation of surgical intervention is important to improve outcomes in patients with cardiac rupture after AMI. Although there are no effective pharmacological strategies for preventing cardiac rupture, Gong et al. [16] conducted a meta-analysis to review four randomized control studies with 68,842 subjects, and found that beta-blockers reduced the risk of cardiac rupture in patients with AMI.

The results of our study would be very useful when clinicians are faced with a cardiac rupture. In addition to AMI, cardiac rupture has also been reported to be associated with conditions such as takotsubo cardiomyopathy [17], infection [18], blunt trauma [19], ventriculography [20], and Swan–Ganz catheterization [21]. Our study notifies clinicians of the possibility that prompt surgery may contribute to an improved prognosis. The frequency of each of these conditions is so low that it is difficult to perform studies to prove the efficacy of a repair surgery. Percutaneous closure of a ruptured left ventricle with an Amplatzer muscular ventricular septal defect occluder may be an alternative, particularly in patients at a high operative risk [22]. Further research is warranted to reduce the risk of death in patients with cardiac rupture.

A limitation of this study was the inability to include patients with cardiac arrest prior to the diagnosis of MI or to include patients with MI without cardiac rupture. Thus, this study is subject to a selection bias. However, cardiac rupture after AMI is a very rare condition, and the registry data in the present study was very valuable. In the future, a nationwide registry may solve this problem [23]. The present study did not examine the association between the cardiac rupture types, including recurrent cardiac rupture, and in-hospital mortality. The clinical characteristics and risk factors of in-hospital mortality in patients with cardiac rupture have already been reported [8]. Furthermore, we have not considered the door-to-balloon time, which is an important factor in in-hospital mortality [24]. In the present study, the primary focus was the association between cardiac rupture time and the risk of death.

## Conclusion

The AMI-to-cardiac rupture time contributes significantly to the risk of in-hospital mortality; however, rapid diagnosis and prompt surgical interventions are crucial for improving the outcomes in patients with cardiac rupture after AMI. The results of this study may provide important insights into the prognostic value of the clinical indicators associated with AMI. Further studies are needed to reduce the risk of death in patients with cardiac rupture.

## Data Availability

The dataset is restricted and not public.
